# UAE Women’s Knowledge and Attitudes towards Physical Activity during Pregnancy

**DOI:** 10.3390/ijerph20176669

**Published:** 2023-08-29

**Authors:** Sharifa AlBlooshi, Rafiq Hijazi, Lynne Kennedy, Ala Al Rajabi

**Affiliations:** 1 Department of Health Sciences, College of Natural and Health Sciences, Zayed University, Dubai P.O. Box 19282, United Arab Emirates; sharifa.alblooshi@zu.ac.ae; 2 Department of Mathematics and Statistics, College of Natural and Health Sciences, Zayed University, Abu Dhabi P.O. Box 144534, United Arab Emirates; rafiq.hijazi@zu.ac.ae; 3 Department of Health Sciences, College of Natural and Health Sciences, Zayed University, Abu Dhabi P.O. Box 144534, United Arab Emirates; ala.rajabi@zu.ac.ae; 4 Department of Human Nutrition, College of Health Sciences, Qatar University, Doha P.O. Box 2713, Qatar

**Keywords:** physical activity (PA), United Arab Emirates, women, knowledge, attitudes, mothers, pregnancy

## Abstract

The benefits of being physically active during pregnancy are widely acknowledged. It is important for the prevention of chronic diseases and the promotion of good health for mothers and children. However, physical activity by women in the UAE is notoriously low and reduced further during pregnancy. The same can be said regarding research about the knowledge and understanding of the benefits and risks associated with exercise as a predictor of behavior. We aimed to assess knowledge and attitudes towards physical activity during pregnancy amongst Emirati women. A cross-sectional digital survey was designed to assess knowledge and attitudes and distributed to women, aged 18–40 years, using non-randomized, purposeful snowball sampling. A total of 1538 women were recruited. Most participants were aged 20–29 years (53.5%), were Emiratis (88.9%), and had no history of chronic disease (68.6%). The participants self-reported very low levels of PA (75.5%) and had a below-average level of knowledge overall (40.6 ± 20). Younger ages (*p* < 0.001), lower educational levels (*p* = 0.004), being employed (*p* = 0.014), and having a history of chronic disease (*p* = 0.016) were significantly associated with lower mean knowledge scores, while being married (*p* = 0.003) was significantly associated with higher scores. The participants also exhibited a positive attitude towards physical activity during pregnancy by selecting answers that they supported it. To encourage physical activity, women living in the UAE could benefit from clear advice about safe physical activity during pregnancy.

## 1. Introduction

The benefits of physical activity (PA) during pregnancy are clear. Remaining physically active during pregnancy can help reduce backaches, relieve constipation, improve heart health, and aid in maintaining a healthy weight [1]. In contrast, physical inactivity during pregnancy is reportedly linked to several risk factors and adverse health outcomes for mother and baby; being inactive increases the risk of maternal obesity, which has been associated with increased risk of premature birth, emergency cesarean sections, and preeclampsia [2,3]. In addition, gestational diabetes mellitus (GDM), a condition in which hormones from the placenta block the body from successfully utilizing insulin, is more likely to develop in pregnant women who are physically inactive. GDM increases the women’s chance of having a c-section, experiencing severe respiratory problems that may cause complications for surgery, and having greater future risk of acquiring type 2 diabetes [3]. The prevention of GDM is critical in the UAE, a region with one of the highest and still rising levels of type 2 diabetes amongst the adult population and above-average GDM [4].

According to both the WHO and the American College of Obstetricians and Gynecologists (ACOG) guidelines, all pregnant and postpartum women without contraindication should aim for at least 150 min of moderate-intensity physical activity each week, even if they were inactive before pregnancy [1,5]. The advice varies depending on prior fitness, general health, and participation in physical activity [2]. Current guidance on physical activity during pregnancy recommends that women who are determined by a physician to be fit and healthy and were physically active before their pregnancy should remain active at safe levels during the three trimesters. Raising awareness and knowledge about the importance of physical activity for pregnant women is of great importance to help minimize health risks to mothers and their children. 

The majority of women are aware that exercise and physical activity is encouraged during pregnancy, and yet despite this most do not partake in or actively avoid physical activity whilst pregnant. Several studies have shown that despite being aware of the benefits of exercise, most women lack information or confidence in their knowledge about the risks associated with physical inactivity during pregnancy [6,7,8,9,10,11].

Furthermore, some have suggested that physical activity decreases further or stops entirely with pregnancy [6,7,12,13]. In the past 50 years, following the discovery of oil, the UAE has transitioned from the Bedouin culture based on pastoralism and farming, traditional to the Middle East and North African (MENA) region, to a wealthy, highly industrialized, and heterogeneous society. Though traditional cultural values and pastimes remain strong, most Emiratis no longer engage in manual work, have hired help, and follow modern, sedentary lifestyles in the cities. Though research into the physical activity levels of Emiratis is scant, surveys have shown that daily PA levels are very low. Even less is known about the PA levels of pregnant women [14,15,16,17,18]. 

Research has suggested that limited or lack of knowledge or confidence in and interpretation of the scientific guidelines for exercising safely during pregnancy may be important factors mediating PA levels amongst pregnant women [11,12,13,14,15,16]. For most pregnant women, there is no additional risk of miscarriage from engaging in regular physical activity during an uncomplicated pregnancy [2,15]. Women have expressed a lack of detailed understanding of the advice, for example, regarding the types of exercise considered safe and suitable during pregnancy [12]. Theoretical models, such as the Theory of Planned Behavior (TPB), have helpfully been used to shed light on this important contradiction in studies of PA behavior among pregnant women, albeit in non-Arab populations. Several studies highlighted intention to be physically active as a strong predictor of PA-related behavior and attitude as a strong predictor of intention [17,18,19]. These studies also concluded that women adopted more positive attitudes, subjective norms, and intentions towards resting, i.e., increasingly sedentary behavior, as their pregnancy progressed [17,18]. Thus, interventions that encourage regular PA may want to focus on early antenatal periods or earlier.

Furthermore, several factors were found to influence women’s motivation or willingness to participate in physical activity during pregnancy. Firstly, there is the perceived difficulty or ease of participating in regular exercise while pregnant. Secondly, there are the social norms of the community they live in. Finally, women who had been participating in regular physical activity prior to their pregnancy were more likely to continue exercising while pregnant [19]. Understanding women’s attitudes, opinions, beliefs, and knowledge about PA during pregnancy as well as encouraging a supportive community may assist in the development of effective interventions promoting PA during pregnancy. 

With many women acknowledging that regular PA is beneficial for health, they also believe that physical activity may increase complications and the risk of miscarriage [13]. For most pregnant women, the actual risk of miscarriage from engaging in ‘regular’ physical activity is relatively low [2]. However, perceived risk is likely to be relatively high due to the perceived severity of the outcome, i.e., miscarriage and loss, and this may therefore inhibit any risk-taking behavior during pregnancy. Downs and Hausenblas [17] and Newham et al. [18] concluded that women in their respective studies adopted more positive attitudes, subjective norms, and intentions towards resting, i.e., increasingly sedentary behavior, as their pregnancy progressed. Additionally, attitudes towards PA during pregnancy were the strongest determination of intention. Thus, interventions that encourage regular PA may want to focus on early ante-natal periods or earlier. Understanding the attitudes, opinions, beliefs, and knowledge about PA during pregnancy may assist in the development of effective interventions promoting PA during pregnancy.

We know that social and cultural traditions are resistant to short- and medium-term change and are therefore more difficult to influence and change [20]. Notwithstanding this, providing greater clarity in the advice around physical activity and safety guidelines may realistically be achieved and help increase women’s knowledge and confidence to be physically active during pregnancy. In countries and regions where levels of physical activity amongst women are already below the recommended levels, avoiding further reductions due to pregnancy is an important public health goal. For women already physically active, greater clarity of the advice around safe exercising during pregnancy may have additive benefits, helping improve maternal and child health. To inform such developments, we first need to clearly understand women’s current levels of knowledge and attitudes towards physical activity during pregnancy in Arab countries such as the UAE. This study aims to investigate the knowledge and attitudes of women living in the UAE, towards physical activity during pregnancy, to assess the need for targeted interventions. 

## 2. Materials and Methods

### 2.1. Study Design

This study aims to investigate how much women living in the UAE know about physical activity during pregnancy in terms of both risks and benefits and their attitudes on whether or not they are willing to participate in physical activity during pregnancy. This knowledge can be used to assess the need for targeted interventions. We have conducted a quantitative cross-sectional study across the seven Emirates of the country: Abu Dhabi, Dubai, Sharjah, Ajman, Umm Al Quwain, Fujairah, and Ras Al Khaimah, and the data were collected from August 2021 to December 2021. 

### 2.2. Sampling

In this study, we have recruited 1537 women from different emirates in the UAE; we used non-probability convenience snowball sampling; and data were collected using an electronic survey that was distributed to the participants via email, WhatsApp, Instagram, and Snapchat. The inclusion criteria were being female, capable of proving consent, able to read and write in English or Arabic, living in the UAE, and being 18 years and older. The exclusion criteria were women below 18 years of age who are not living in the UAE and those who declined participation. Having a previous or chronic health condition was not an exclusion criterion.

### 2.3. Data Collection

Data were collected through a structured electronic survey. The questions regarding the physical activity knowledge were adapted from a self-administered questionnaire, tested, and validated earlier then modified to match the study’s purposes taking into consideration the WHO guidelines [21,22]. The PA and exercise were defined to the participants as per the WHO guidelines “bodily movement produced by skeletal muscles that results in energy expenditure” [5]. A semi-structured online (digital) questionnaire was designed using Google Forms, to collect data. The survey consisted of 20 questions in two parts. The first included (*n* = 9) questions regarding socio-demographic data (e.g., age, education level, marital status, nationality, age, health status, and employment status). The second part included (*n* = 11) questions regarding knowledge, attitude, and practice of physical activity (e.g., benefits of physical activity during pregnancy, types, harmful, and situational barriers to physical activity). It was initially created in English before being translated into Arabic; then, it was reviewed by the authors and pilot-tested on 20 participants for face validity.

### 2.4. Data Analysis

Statistical analysis was performed using IBM SPSS Statistics 28 (IBM, Armonk, NY, USA). Frequencies and percentages were used to summarize demographic, health-related, and physical activity variables. Independent samples t-test and One-Way Analysis of Variance (ANOVA) were utilized to compare participants’ knowledge on physical activity during pregnancy based on their demographic characteristics. A multiple linear regression model was developed to identify the factors associated with the physical activity knowledge of participants. Finally, the Chi-Square Test of Independence was used to test the association of demographic, health, and physical activity characteristics of participants with their attitudes toward physical activity during pregnancy. *p*-Values less than 0.05 were considered statistically significant.

A knowledge index was created using the 14 knowledge items. All items were given equal weight, and for each item, the correct response was coded as 1, while the wrong one was coded as 0. The response codes for each woman were added to generate a score for that woman, and the score ranged from 0 to 14 as there were fourteen knowledge items. The results were calculated out of 100 after converting it to a 100 percent scale for more effortless reading by dividing the score of each item by 14 and then multiplying the result by 100.

The regression model was used to further analyze the correlation of the knowledge about PA during pregnancy among women in UAE. The model was developed using potential factors such as current PA activity and medical history while controlling for sociodemographic characteristics of the participating women.

### 2.5. Ethical Considerations

The study was designed and implemented according to the ethical standards of Zayed University. Ethical approval was granted by the College of Natural and Health Sciences (CNHS), Ethics Application Code ZU21_061_F. Before starting the survey, participants were provided with an informed consent form that clarified the purpose of the study, what was expected of the participants, and contact information. Participants were advised that the survey was voluntary and that they had the right to withdraw at any stage of the study. Confidentiality and anonymity were assured throughout the study. Participants were not harmed during the study process, and no significant ethical issues resulting from participation were identified. 

## 3. Results

### 3.1. Demographic Characteristics

The questionnaire was completed by 1537 women aged 18–40 years, residing in the United Arab Emirates. Table 1 displays the women’s demographic characteristics. Most (53.5%) of the participants were aged 20–29 years, were Emiratis (88.9%), were residing in Abu Dhabi (48.1%), were residing in urban areas (88.0%), were married (82.1%), held a bachelor’s degree (40.3%), and were unemployed (59.7%).

### 3.2. Medical History and Physical Activity

Table 2 shows that most participants had no history of chronic disease at 68.6%. The most common chronic diseases were migraine headaches (7.6%) and diabetes (7.1%). Other chronic diseases including cardiovascular disease, cancer, hypertension, kidney disease, arthritis, parathyroid, hypothyroid, gastrointestinal disease, asthma, and respiratory disease affected between 2.0% to 5.7% of participants. Most of the participants reported various levels of physical activity: 1–2 times/week (40.5%), 3–5 times/week (19.5%), and daily (5.4%), while 35.0% of participants reported being inactive or rarely active. In addition, among the participants, 23.8% were pregnant, and 13.3% were breastfeeding. 

### 3.3. Participants’ Knowledge toward Physical Activity during Pregnancy

To assess knowledge on physical activity during pregnancy, participants were asked two “select all that apply” questions (Recommended physical activity during pregnancy. Benefits of physical activities during pregnancy) and a single “agree/disagree” question (Some types of physical activity should be avoided during pregnancy; Table 3). All options included in the “select all that apply” questions and the “agree” response were correct.

Table 3 lists frequencies and percentages of participants who selected the correct response to a question. When asked “Which type of physical activity is recommended during pregnancy”, 80% of participants selected walking; 31% selected swimming; 11% selected running; and only 1% selected yoga. A total of 78% agreed that some types of physical activity should be avoided during pregnancy.

In addition, many participants believed that physical activity during pregnancy decreases the risk of gestational diabetes (60%), enhances the cardiovascular system (51%), and strengthens the musculoskeletal system (50%). In addition, less than half agreed that physical activity during pregnancy decreases the risk of gestational hypertension (44%), provides better sleeping patterns (41%), helps manage weight gain (38%), and improves respiratory function (37%). Finally, only 25% believed that it enhances the endocrine and gastrointestinal systems, and 21% believed it increases amniotic fluid.

### 3.4. Knowledge Index among Women in the UAE

As per the literature review, it has been demonstrated that a scoring system for the questions that assess participants’ knowledge of physical activity best suits this study. A knowledge index was created using the 14 knowledge items in Table 3. 

Overall, the mean knowledge of the participants was 40.6 ± 20.1 with the maximum score being 92.9 and the minimum score being 7.1. This indicates a below-average level of knowledge overall. Table 4 shows the mean knowledge index scores according to demographic characteristics. Older age (*p* < 0.001), being married (*p* < 0.001), higher educational attainment (*p* < 0.001), and absence of chronic disease history (*p* = 0.004) are all significantly associated with higher knowledge index scores.

The regression model was used to further analyze the correlates of the knowledge about PA during pregnancy among women in the UAE. The model was developed using potential factors such as current PA activity and medical history while controlling for sociodemographic characteristics of the participating women. The results are illustrated in Table 4. Being younger in age (*p* < 0.001), having a lower educational level (*p* = 0.004), being employed (*p* = 0.014), and having a history of chronic disease (*p* = 0.016) were significantly associated with lower mean knowledge score, while being married (*p* = 0.003) was significantly associated with higher scores.

### 3.5. Attitudes toward Physical Activity among Women during Pregnancy

Table 5 shows the attitudes of participants toward physical activity during pregnancy. Overall, participants exhibited a positive attitude toward physical activity during pregnancy. A total of 90.9% of the participants agreed that physical activity benefits both the mother and the child. Some 80.8% agreed that mothers without pregnancy complications should begin PA program during pregnancy. Furthermore, 85.6% concurred that pregnant women who used to be physically active should be encouraged to continue an exercise program throughout pregnancy. On the other hand, 69.7% disagreed that physical activity during pregnancy increases the risk of low-birth-weight babies. Finally, 70.7% concurred that the possible harmful effects of physical activity during the pregnancy on the fetus are minimal or non-existent.

Each attitude item was compared to demographic characteristics, as seen in Table A1, Table A2, Table A3, Table A4 and Table A5. In Table A1, regarding the statement “Physical activity during pregnancy is beneficial for both mothers and infants”, those who were older women (*p* < 0.001), were married (*p* < 0.001), had a high education level (*p* < 0.001), and had no chronic diseases (*p* < 0.001) were more likely to strongly agree or agree with this statement. In Table A2, considering the statement “A woman without complications during pregnancy should not begin an exercise during pregnancy”, women from Abu Dhabi (*p* < 0.001) were more likely to strongly agree or agree with this statement.

In Table A3, about the statement “Pregnant women who used to exercise should be encouraged to continue an exercise or PA program throughout pregnancy”, those who were older women (*p* < 0.001), were from Abu Dhabi (*p* < 0.001), were married (*p* < 0.001), had a high education level (*p* < 0.001) and had no medical history (*p* = 0.001) were more likely to strongly agree or agree with the statement. 

In Table A4, concerning the statement “Physical activity during pregnancy increases the risk of a low birth weight”, those who were unmarried (*p* < 0.001), were employed (*p* = 0.001), and had a high education (*p* = 0.001) level were more likely to strongly agree or agree with this statement. In Table A5, regarding the statement “The possible harmful effects of physical activity during the pregnancy on the fetus are minimal or non-existent”, women from Abu Dhabi (*p* < 0.001) were more likely to strongly agree or agree with this statement.

## 4. Discussion

The present study has examined current levels of awareness, knowledge, and practice regarding safe engagement in physical activity during pregnancy among Emirati women living in the UAE. Since it is widely accepted that many factors mediate KAP (Knowledge, Attitude, and Practices), studies aimed at understanding or moderating KAP should also be socially and culturally situated. To the best of our knowledge, this is one of two published studies on this issue in this population group and social and cultural context. Furthermore, this study is the first to include women from all seven Emirates of the UAE. Data were collected from 1537 women who completed the online survey, and the majority (88.9%) self-identified as Emirati, which is a key strength of the current paper, with most of the women (80%) residing in Abu Dhabi (48%) or Dubai (32.3%), the two most populated of the seven Emirates. The remaining 20% were recruited from the minor emirates; nearly all respondents (88%) lived in urban as opposed to rural areas. As the section below indicates, we were able to describe differences according to pregnancy; however, future studies might also collect details about parity and trimester of pregnancy to enable us to examine the influence of this on KAP. 

### 4.1. Physical Activity Levels

Adopting regular physical activity during pregnancy improves health and well-being, results in safer birthing, and protects against excessive gestational weight gain, avoiding manifestations such as gestational hypertension or diabetes [5]. Women participating in the present study self-reported very low levels of PA. The majority (75.5%) rated their current activity as ‘rarely’, i.e., occasional (35%) or only 1–2 times/week (40.5%), and only 1 in 20 (5.4%) women reported daily engagement in PA, suggesting as a population, women residing in the UAE are extremely sedentary and inactive. Our findings support recent surveys indicating that physical activity levels are low in the Arab region, including the UAE [13,14,15]. This situation has not changed considerably over time, as indicated by earlier surveys of PA undertaken in the UAE [23,24,25]. Reports also suggest that levels tend to be particularly low among women [13,14,15,25]. Moreover, as the study of PA knowledge and practice among Emirati women living in Dubai [26] reported, the majority (87.9%) of the women in their study were classified, based on objective assessment using the validated PPAQ, as engaging in light sedentary activity, i.e., being inactive; moreover, sitting or watching TV were cited as the women’s preferred activity, especially when pregnant.

International guidance strongly recommends that most women should continue with or engage in regular physical activity (PA) during pregnancy, and those who previously participated in sports, exercise, or regular physical activity should try to maintain this under the supervision and guidance of their physician or maternity care providers, since most forms may be continued safely. Despite this recommendation, our findings suggest that participation in regular PA by Emirati women may decline or stop completely with pregnancy. We also posit that women in the UAE are becoming increasingly sedentary. This claim is supported by a recent objectively quantified study of physical activity and sedentary behavior in a young UAE population [15]. Thus, as energy expenditure is drastically reduced and energy intake, especially during pregnancy, continues to exceed requirements, women in the UAE are likely to face rising risks of gestational obesity and its complications.

### 4.2. Factors Influencing PA

In order to influence and encourage regular PA amongst Emirati women, a better understanding of why PA levels are low is needed. With respect to all lifestyle behavior, levels of PA are compounded by specific social and cultural factors [26].

A high proportion (83.7%) of the women in our sample were aged between 18 and 40 years and of reproductive age, with most women aged between 20 and 29 years (53.5%) with 23.4% aged 30–39 years. Also, most women were married (82%), representing local cultural traditions. Since the UAE was established in 1971, it has undergone rapid social and economic change, facilitated by many successful government initiatives designed to protect the indigenous population and promote Emiratization, including the promotion of women in the workplace and participation in higher education. Thus, not surprisingly, many (40%) of the women surveyed reported being in full or part-time employment, with those women who are studying included in the unemployed (59.7%) (studying, retired, or staying at home), whilst overall, a relatively high proportion of respondents (40%) were in receipt of a high school degree or bachelor’s degree. Although cultural traditions in support of marriage appear to remain strong in this region, only a third were currently pregnant or lactating (23.8% self-identified as pregnant with a further 14% breastfeeding), suggesting that whilst marriage remains traditional and valued in the UAE, this is no longer as strong a predictor of childbirth. As national statistics highlight, the number of births per family has and continues to fall sharply [27]. So, although birth rates are decreasing, the individual and societal costs of sedentary behavior amongst pregnant women are rising, placing an unnecessary burden on the health system. Furthermore, despite this, a recent study on UAE women [26] reports that educational attainment, age (i.e., being younger), and parity (having fewer children) may still positively mediate PA during pregnancy in this region. Since marriage, parity, and education appear to mediate PA practice amongst women during pregnancy, future research might focus on better understanding the role of socio-demographic factors over time and in this rapidly changing context.

Our study points to low levels of physical activity amongst Emiratis during pregnancy and is consistent with published reports claiming Arab and UAE populations, especially women, are disinterested in exercise and prefer sedentary practices [14,27,28,29]. Similarly, a recent systematic review of the contextual barriers to PA during pregnancy [30], undertaken in the Middle East and North African (MENA) region, also emphasized the importance of environmental factors, including lack of time (due to homemaking), absence of social support, tiredness, and lack of knowledge and information from healthcare providers as relevant factors mediating PA generally and during pregnancy in a similar geographic area. However, since our study was not aimed at exploring specific factors hindering inactivity, we refer to relevant research undertaken in the region to interpret our findings.

Regardless of positive behavioral intention, low levels of PA among women in the region can be attributed to the inhospitable climate, which prevents people from being active and increases sedentary behavior (labor, transportation, or leisure time), especially outdoors. A comprehensive systematic review of PA levels reports that the UAE climate is a major barrier to PA, especially the intense heat of the summer months (June–September), when outdoor temperatures exceed 40 degrees Celsius [28]. Others indicate gender-specific barriers; for example, the lack of female-only facilities [14,15,27,28,29]. This concurs with studies of attitudes towards PA amongst women living in the region (e.g., [7,13,24,29]).

It should be noted that the UAE is a country of rapid economic and social transition. Since 1971, the UAE has experienced unprecedented industrialization, and the social context has changed considerably; therefore, earlier studies into the barriers and enabling factors (i.e., Geeber and Person [30]) may no longer be relevant. For example, the general accessibility and affordability of opportunities to engage in PA, even during the summer, has increased dramatically, as the number of indoor and air-conditioned gyms and walking and cycling arenas has increased beyond recognition. Dress codes have also relaxed including sportswear designed to meet modesty and Hijab (faith) requirements, whilst attitudes towards PA appear to be changing for the better, especially since major sporting events such as the 2022 World Cup have been increasingly hosted in this region. This is consistent with the study by Harrison and colleagues [31], in their systematic review of attitudes towards PA during pregnancy. Further research, especially qualitative studies, is needed to better understand the social and cultural nuances associated with barriers and enabling factors for PA amongst pregnant Emirati women. Future health promotion campaigns for awareness raising or health education might usefully target younger, unmarried, and employed groups. This recommendation resonates with other research, whereby gender-specific social marketing campaigns may be more effective in overcoming real or perceived lack of support or accessibility to PA facilities and cultural norms that place low value on female participation in sports and physical activity [31,32,33]. Consistent with other research, we acknowledge the increasing attention on the role of the physical environment. In particular, accessing opportunities for PA is very important for encouraging increased participation in PA, especially the engagement of females and women of childbearing age in this region. Special consideration on the cultural values of this region, and the climate should also be considered [34] when policies aimed at increasing PA levels are developed. 

### 4.3. Knowledge of the Health Benefits of PA

It is widely acknowledged that knowledge and beliefs relating to the benefits or risks associated with health-related behavior positively mediate behavioral intention and practice. When asked whether regular exercise or PA was safe during pregnancy, one-third (36.6%) agreed it was ‘not harmful’, i.e., safe. When asked about which type of physical activity is recommended during pregnancy, the majority (78%) agreed that ‘some’ forms of physical activity should be avoided during pregnancy, while walking (80%), swimming (31%), and running (11%) are, however, acceptable. Only 1% of women mentioned yoga as safe to undertake during pregnancy, although this is not surprising given that it is not common or widespread in the UAE. According to the American College of Obstetricians and Gynecology (ACOG), walking, swimming, and yoga are the safest options for PA during pregnancy and, moreover, can easily be incorporated into most lifestyles, whereas high-intensity activity, contact sports, scuba diving, and activity with a high risk of abdominal trauma are unsafe and should be avoided [2]. Internationally recognized authorities also approve and promote yoga (e.g., ACOG) [2]. Therefore, future campaigns promoting PA during pregnancy in the UAE might specifically encourage greater awareness about its safety and health benefits. 

Many of our participants believed in the health benefits of physical activity during pregnancy, specifically by helping to decrease risks of developing gestational diabetes (60%), which may improve health associated with the cardiovascular system (51%) and improve musculoskeletal strength (50%). Additionally, almost half agreed that physical activity during pregnancy decreases the risk of gestational hypertension (44%), provides better sleeping patterns (41%), helps avoid gestational weight gain (38%), and improves respiratory function (37%), while 25% believed it might enhance health associated with endocrine and gastrointestinal systems. This concurs with a study of the knowledge and participation in exercise and physical activity among pregnant women in Ghana [35]. Of the total participants (*n* = 77) recruited, 57 (74%) scored high in PA knowledge, with the majority of 48 (62.3%) agreeing with the statement that PA promotes healthy pregnancy. 

Compared with other published studies, the overall mean index score for knowledge of 40% (cf. 92% max score; SD 20%) is lower than expected. This contradicts previously published studies, albeit in other social and cultural contexts, whereby relatively high levels of knowledge and awareness of the guidance and professional advice around PA during pregnancy have been reported. For example, Alaglan reported in a study conducted in Saudi Arabia that the majority of women had high knowledge levels (mean = 77; median = 75) about types and amounts of physical activity and they thought that physical exercise during pregnancy was necessary [36]. Similarly, a study of physical activity during pregnancy and the state of Polish women’s knowledge reported that the majority (92.5%) of women were aware of the benefits of physical activity during pregnancy, and 80.0% and 82.4% of the participants were identified as aware of the reduced risks of GDM and preeclampsia, respectively, which was positively associated with their physical activity. Moreover, greater awareness was associated with higher educational attainment (*p* = 0.001) [37].

As mentioned, overall the mean knowledge scores in our sample are lower than expected. Regression analysis for knowledge and socio-demographic data indicates a significant positive association between knowledge scores and age, marital status, educational attainment, employment status, and having a chronic condition. Although consistent with similar research based on the lower-than-expected levels of knowledge reported, this may be exacerbated for women who are younger, i.e., below 20 years, become pregnant in their teen years (*p* < 0.001), are of lower educational attainment (high school) (*p* = 0.004), are employed (*p* = 0.014), or have a history of chronic disease (*p* = 0.016), since these factors were significantly associated with lower mean knowledge score. Conversely, being married (*p* = 0.003), of higher social status, and being of higher age may be slightly protective, in that these were significantly associated with higher knowledge scores. Thus, future planning for awareness raising or health education campaigns might usefully target younger, unmarried, unemployed, and student sub-groups. Since the majority of our respondents were Emirati (89%), were of childbearing ages (78% aged 20–29; 30–39), and had low overall knowledge index scores, policymakers might consider the need for more specific campaigns to raise awareness and educate women living in the UAE on the importance of regular and safe forms of PA during pregnancy. Furthermore, utilizing social marketing techniques to specifically target a relatively small and dispersed female Emirati population, to capture all ages, social statuses, and marital statuses, is also recommended to take into account specific social and cultural nuances in this diverse and dispersed population.

Further research might also be warranted to establish knowledge and awareness levels, including health and physical activity literacy and the various levels of ability to translate health information more precisely into practice. Furthermore, approximately 1:5 women (21%) believed that amniotic fluid and thus the health of the unborn are adversely affected by physical activity [38,39]. More research into the health beliefs of Arab women, especially in the Gulf Cooperation Council (GCC) countries, and carefully targeted messages that acknowledge alternative belief systems are recommended for this region. Finally, since knowledge and attitudes are essential predictors or mediators of behavior, including PA, greater involvement of midwives and physicians in promoting safe PA is highly recommended. As previous research shows, some professionals lack confidence in raising issues of diet, PA, or gestational weight gain with mothers to be, for fear of upsetting them or undermining their confidence, and therefore professionals involved with childbirth should receive adequate training [40].

### 4.4. Limitations of the Study

Self-reported rating of physical activity level was used in the present study, and since we did not quantify physical activity level, consequently, we were unable to assess the percentage of participants that meet global recommendations (150 min/week) for the general population. In terms of validity, we asked participants to qualitatively assess their physical activity levels, which in hindsight is less reliable than objective assessments; however, as a cross-sectional survey with limited resources and time, we acknowledge the shortcomings and suggest caution when interpreting findings relating to the activity. 

Another limitation is that this study did not further investigate the parity/gravidity, the status of the participants in terms of former pregnancies, the status of current pregnancies, the number of months ante-natal or post-natal, the number of children, and knowledge of how many were mothers, as it could it just be that some may not have engaged with any thought or material around PA during pregnancy. Furthermore, considering the socio-demographic variables associated with PA levels would have provided a better understanding of behavior among women in the UAE.

Through addressing gaps in the understanding of the association between knowledge and practice relating to PA during pregnancy in this region, this study provides additional support for designing interventions tailored towards women, especially Emirati women. 

## 5. Conclusions

Our findings suggest that most Emirati women are aware of the health benefits of physical activity during pregnancy, and yet despite this, few women of reproductive age participate in PA, and this appears to decline further during pregnancy and lactation. Furthermore, knowledge surrounding the safety of physical activity and exercise is lacking. Given the health benefits of physical activity during pregnancy, especially in helping prevent gestational diabetes and related complications, women living in the UAE could benefit from clear advice about safe physical activity during pregnancy. Additionally, more research utilizing behavioral theories to help model practice, including the intention to participate (in PA), may increase understanding of how PA behavior is mediated in this socio-cultural context. Understanding women’s knowledge and beliefs will help inform future interventions or messaging campaigns and demands a culturally relevant approach.

## Figures and Tables

**Table 1:** Demographic information of the study population (*n* = 1537 women).

	Categories	Frequency	Percentage
Age (years)	18–20	105	6.8%
	20–29	821	53.5%
	30–39	359	23.4%
	40+	251	16.3%
Nationality	Non-Emirati	170	11.1%
	Emirati	1367	88.9%
Emirate	Abu Dhabi	739	48.1%
	Dubai	496	32.3%
	Northern Emirates	302	19.6%
Residential area	Rural	184	12.0%
	Urban	1353	88.0%
Marital status	Married	1262	82.1%
	Divorced/Widowed/Separated	275	17.9%
Educational level	Less than High School	97	6.3%
	High School Degree	541	35.2%
	Bachelor’s degree	787	51.2%
	Master or PhD Degree	112	7.3%
Employment status	Employed (Full-time, Part-time, Self-employed)	620	40.3%
	Unemployed (Student, Retired, Housewife)	917	59.7%

**Table 2:** Medical history and physical activity among study population (*n* = 1537 women).

Category	Frequency	Percentage
Chronic diseases	*N*	%
Cardiovascular diseases	30	2.0%
Diabetes	109	7.1%
Cancer	43	2.8%
Asthma and respiratory diseases	87	5.7%
Hypertension	79	5.1%
Kidney diseases	34	2.2%
Arthritis	55	3.6%
Migraine headaches	117	7.6%
Parathyroid	36	2.3%
Hypothyroid	76	4.9%
Gastrointestinal diseases	69	4.5%
Has a medical history of chronic disease	*N*	%
No	1054	68.6%
Yes	483	31.4%
Current physical activity	*N*	%
Never and rare	538	35.0%
1–2 times per week	623	40.5%
3–5 times per week	293	19.1%
Daily	83	5.4%
Breastfeeding or Pregnant	*N*	%
Breastfeeding	205	13.3%
Pregnant	366	23.8%
Neither	864	56.2%

**Table 3:** Participants’ knowledge about physical activity during pregnancy.

Categories	Frequency	Percentage
Recommended physical activity during pregnancy	*N*	%
Swimming	480	31%
Walking	1222	80%
Running	176	11%
Yoga	17	1%
Some types of physical activity should be avoided during pregnancy	1200	78%
Benefits of physical activities during pregnancy (tick more than 1)	*N*	%
Weight gain management	578	38%
Cardiovascular system enhancements	778	51%
Musculoskeletal improvements and bone strengthening	772	50%
Better sleeping patterns	632	41%
Decreased risk of gestational diabetes	924	60%
Improves respiratory function	564	37%
Decreased risk of gestational hypertension	679	44%
Enhance Endocrine and gastrointestinal system	387	25%
Increased amniotic fluid	327	21%

**Table 4:** Regression analysis of knowledge index by demographic characteristics.

		Unadjusted Comparison ^a^			Regression ^b^	
	*N*	Mean	SD	*p*-Value	Coefficient	*p*-Value
Age (years)						<0.001
18–20	105	34.35	19.51	<0.001	−10.34	<0.001
20–29	821	39.93	19.82		−5.67	<0.001
30–39	359	41.35	20.07		−3.70	0.025
40+	251	44.39	20.79		Ref	
Nationality						
Emirati	1367	40.93	20.21	0.064	1.76	0.281
Non-Emirati	170	37.90	19.36		Ref	
Emirate						0.787
Abu Dhabi	739	40.77	19.96	0.603	0.77	0.574
Dubai	496	40.97	20.66		1.00	0.500
Northern Emirates	302	39.57	19.70		Ref	
Residential Area						
Urban	1353	40.70	20.17	0.581	−0.25	0.874
Rural	184	39.83	19.90		Ref	
Marital status						
Married	1262	41.67	20.01	<0.001	4.19	0.003
Divorced/Widowed/Separated	275	35.66	19.99		Ref	
Educational Level						0.004
Less than High School	97	33.51	17.35	0.001	−8.13	0.004
High School Degree	541	39.64	19.81		−2.21	0.307
Bachelor’s Degree	787	41.91	20.33		−0.42	0.837
Masters or PhD Degree	112	42.16	21.20		Ref	
Employment status						
Employed (Full-time, Part-time, Self-employed)	620	39.55	20.10	0.093	−2.70	0.014
Unemployed (Student, Retired, Housewife)	917	41.31	20.13		Ref	
Medical History						
Yes	483	38.42	20.12	0.004	−2.72	0.016
No	1054	41.60	20.06		Ref	
Current physical activity						0.107
Never and rare	538	39.95	19.96	0.188	−4.86	0.038
1–2 times per week	623	40.11	19.70		−4.12	0.076
3–5 times per week	293	41.78	20.57		−2.39	0.332
Daily	83	44.32	22.55		Ref	

^a^. The associations between participants’ knowledge on physical activity during pregnancy with their sociodemographic characteristics were assessed by using independent samples *t*-test (significance at *p* < 0.05). ^b^. The associations between participants’ knowledge on physical activity during pregnancy with their sociodemographic characteristics were assessed by using One-Way Analysis of Variance (ANOVA) (significance at *p* < 0.05).

**Table 5:** Attitudes toward physical activity among women during pregnancy.

	Strongly Agree	Agree	Disagree	Strongly Disagree
	Frequency (%)	Frequency (%)	Frequency (%)	Frequency (%)
Physical activity during pregnancy is beneficial for both mothers and infants	637 (41.4%)	761 (49.5%)	103 (6.7%)	36 (2.3%)
A woman without complications during pregnancy should not begin an exercise program during pregnancy.	172 (11.2%)	123 (8.0%)	855 (55.6%)	387 (25.2%)
Pregnant women who used to exercise should be encouraged to continue an exercise program throughout pregnancy	565 (36.8%)	750 (48.8%)	187 (12.2%)	35 (2.3%)
Physical activity during pregnancy increases the risk of a low birth weight	174 (11.3%)	291 (18.9%)	930 (60.5%)	142 (9.2%)
The possible harmful effects of physical activity during the pregnancy on the fetus are minimal or non-existent	282 (18.3%)	805 (52.4%)	390 (25.4%)	60 (3.9%)

## Data Availability

The data presented in this study are available on request from the first author.

## Appendix A

**Table A1:** First attitude item according to demographic characteristics.

Physical Activity during Pregnancy Is Beneficial for Both Mothers and Infants										
		Strongly Agree		Agree		Disagree		Strongly Disagree		
		*N*	%	*N*	%	*N*	%	*N*	%	*p*-Value
Age	Less than 20 years	34	32.4%	49	46.7%	17	16.2%	5	4.8%	<0.001
	20–29 years	329	40.1%	414	50.4%	57	6.9%	21	2.6%	
	30–39 years	154	42.9%	175	48.7%	23	6.4%	7	1.9%	
	40+ years	120	47.8%	122	48.6%	6	2.4%	3	1.2%	
Nationality	Non-Emirati	74	43.5%	86	50.6%	7	4.1%	3	1.8%	0.490
	Emirati	563	41.2%	675	49.4%	96	7.0%	33	2.4%	
Emirate	Abu Dhabi	326	44.1%	355	48.0%	40	5.4%	18	2.4%	0.323
	Dubai	191	38.5%	256	51.6%	38	7.7%	11	2.2%	
	Northern Emirates	120	39.7%	150	49.7%	25	8.3%	7	2.3%	
Residential Area	Rural	73	39.7%	90	48.9%	15	8.2%	6	3.3%	0.663
	Urban	564	41.7%	671	49.6%	88	6.5%	30	2.2%	
Marital status	Married	547	43.3%	620	49.1%	68	5.4%	27	2.1%	<0.001
	Divorced/Widowed/	90	32.7%	141	51.3%	35	12.7%	9	3.3%	
Educational Level	Less than High School	35	36.1%	40	41.2%	16	16.5%	6	6.2%	<0.001
	High School Degree	219	40.5%	281	51.9%	28	5.2%	13	2.4%	
	Bachelor’s degree	338	42.9%	388	49.3%	49	6.2%	12	1.5%	
	Masters or PhD Degree	45	40.2%	52	46.4%	10	8.9%	5	4.5%	
Employment status	Employed (Full-time, Part-time, Self-employed)	243	39.2%	313	50.5%	44	7.1%	20	3.2%	0.159
	Unemployed (Student, Retired, Housewife)	394	43.0%	448	48.9%	59	6.4%	16	1.7%	
Has a medical history	No medical history	451	42.8%	528	50.1%	58	5.5%	17	1.6%	<0.001
	Medical History	186	38.5%	233	48.2%	45	9.3%	19	3.9%	
Current physical activity	Never and rare	230	42.8%	271	50.4%	27	5.0%	10	1.9%	0.005
	1–2 times per week	251	40.3%	321	51.5%	38	6.1%	13	2.1%	
	3–5 times per week	111	37.9%	141	48.1%	32	10.9%	9	3.1%	
	Daily	45	54.2%	28	33.7%	6	7.2%	4	4.8%	

**Table A2:** Second attitude item according to demographic characteristics.

A Woman without Complications during Pregnancy Should Not Begin an Exercise Program during Pregnancy										
		Strongly Agree		Agree		Disagree		Strongly Disagree		
		*N*	%	*N*	%	*N*	%	*N*	%	*p*-Value
Age	Less than 20 years	8	7.6%	13	12.4%	66	62.9%	18	17.1%	0.048
	20–29 years	92	11.2%	72	8.8%	449	54.7%	208	25.3%	
	30–39 years	38	10.6%	26	7.2%	190	52.9%	105	29.2%	
	40+ years	33	13.1%	12	4.8%	150	59.8%	56	22.3%	
Nationality	Non-Emirati	20	11.8%	15	8.8%	90	52.9%	45	26.5%	0.898
	Emirati	152	11.1%	108	7.9%	765	56.0%	342	25.0%	
Emirate	Abu Dhabi	82	11.1%	78	10.6%	374	50.6%	205	27.7%	<0.001
	Dubai	60	12.1%	29	5.8%	297	59.9%	110	22.2%	
	Northern Emirates	30	9.9%	16	5.3%	184	60.9%	72	23.8%	
Residential Area	Rural	17	9.2%	12	6.5%	98	53.3%	57	31.0%	0.232
	Urban	155	11.5%	111	8.2%	757	55.9%	330	24.4%	
Marital status	Married	151	12.0%	100	7.9%	688	54.5%	323	25.6%	0.117
	Divorced/Widowed/	21	7.6%	23	8.4%	167	60.7%	64	23.3%	
Educational Level	Less than High School	11	11.3%	6	6.2%	53	54.6%	27	27.8%	0.824
	High School Degree	50	9.2%	48	8.9%	302	55.8%	141	26.1%	
	Bachelor’s degree	96	12.2%	59	7.5%	439	55.8%	193	24.5%	
	Masters or PhD Degree	15	13.4%	10	8.9%	61	54.5%	26	23.2%	
Employment status	Employed (Full-time, Part-time, Self-employed)	77	12.4%	51	8.2%	345	55.6%	147	23.7%	0.502
	Unemployed (Student, Retired, Housewife)	95	10.4%	72	7.9%	510	55.6%	240	26.2%	
Has a medical history	No medical history	124	11.8%	88	8.3%	585	55.5%	257	24.4%	0.504
	Medical History	48	9.9%	35	7.2%	270	55.9%	130	26.9%	
Current physical activity	Never and rare	58	10.8%	41	7.6%	290	53.9%	149	27.7%	0.272
	1–2 times per week	68	10.9%	62	10.0%	343	55.1%	150	24.1%	
	3–5 times per week	34	11.6%	16	5.5%	172	58.7%	71	24.2%	
	Daily	12	14.5%	4	4.8%	50	60.2%	17	20.5%	

**Table A3:** Third attitude item according to demographic characteristics.

Pregnant Women Who Used to Exercise Should Be Encouraged to Continue an Exercise Program throughout Pregnancy										
		Strongly Agree		Agree		Disagree		Strongly Disagree		
		*N*	%	*N*	%	*N*	%	*N*	%	*p*-Value
Age	Less than 20 years	25	23.8%	49	46.7%	24	22.9%	7	6.7%	<0.001
	20–29 years	292	35.6%	408	49.7%	100	12.2%	21	2.6%	
	30–39 years	150	41.8%	160	44.6%	44	12.3%	5	1.4%	
	40+ years	98	39.0%	132	52.6%	19	7.6%	2	0.8%	
Nationality	Non-Emirati	66	38.8%	77	45.3%	17	10.0%	10	5.9%	0.006
	Emirati	499	36.5%	673	49.2%	170	12.4%	25	1.8%	
Emirate	Abu Dhabi	292	39.5%	359	48.6%	76	10.3%	12	1.6%	<0.001
	Dubai	158	31.9%	244	49.2%	83	16.7%	11	2.2%	
	Northern Emirates	115	38.1%	147	48.7%	28	9.3%	12	4.0%	
Residential Area	Rural	83	45.1%	68	37.0%	24	13.0%	9	4.9%	<0.001
	Urban	482	35.6%	682	50.4%	163	12.0%	26	1.9%	
Marital status	Married	478	37.9%	624	49.4%	143	11.3%	17	1.3%	<0.001
	Divorced/Widowed/	87	31.6%	126	45.8%	44	16.0%	18	6.5%	
Educational Level	Less than High School	34	35.1%	35	36.1%	24	24.7%	4	4.1%	<0.001
	High School Degree	180	33.3%	274	50.6%	74	13.7%	13	2.4%	
	Bachelor’s degree	305	38.8%	399	50.7%	67	8.5%	16	2.0%	
	Masters or PhD Degree	46	41.1%	42	37.5%	22	19.6%	2	1.8%	
Employment status	Employed (Full-time, Part-time, Self-employed)	224	36.1%	304	49.0%	72	11.6%	20	3.2%	0.213
	Unemployed (Student, Retired, Housewife)	341	37.2%	446	48.6%	115	12.5%	15	1.6%	
Has a medical history	No medical history	400	38.0%	526	49.9%	111	10.5%	17	1.6%	0.001
	Medical History	165	34.2%	224	46.4%	76	15.7%	18	3.7%	
Current physical activity	Never and rare	210	39.0%	262	48.7%	54	10.0%	12	2.2%	0.069
	1–2 times per week	225	36.1%	311	49.9%	78	12.5%	9	1.4%	
	3–5 times per week	99	33.8%	138	47.1%	47	16.0%	9	3.1%	
	Daily	31	37.3%	39	47.0%	8	9.6%	5	6.0%	

**Table A4:** Fourth attitude item according to demographic characteristics.

Physical Activity during Pregnancy Increases the Risk of a Low Birth Weight										
		Strongly Agree		Agree		Disagree		Strongly Disagree		
		*N*	%	*N*	%	*N*	%	*N*	%	*p*-Value
Age	Less than 20 years	16	15.2%	24	22.9%	58	55.2%	7	6.7%	0.074
	20–29 years	100	12.2%	166	20.2%	474	57.7%	81	9.9%	
	30–39 years	40	11.1%	59	16.4%	233	64.9%	27	7.5%	
	40+ years	18	7.2%	42	16.7%	164	65.3%	27	10.8%	
Nationality	Non-Emirati	23	13.5%	28	16.5%	97	57.1%	22	12.9%	0.200
	Emirati	151	11.0%	263	19.2%	833	60.9%	120	8.8%	
Emirate	Abu Dhabi	85	11.5%	147	19.9%	448	60.6%	59	8.0%	0.477
	Dubai	50	10.1%	86	17.3%	306	61.7%	54	10.9%	
	Northern Emirates	39	12.9%	58	19.2%	176	58.3%	29	9.6%	
Residential Area	Rural	26	14.1%	29	15.8%	105	57.1%	24	13.0%	0.100
	Urban	148	10.9%	262	19.4%	825	61.0%	118	8.7%	
Marital status	Married	127	10.1%	223	17.7%	792	62.8%	120	9.5%	<0.001
	Divorced/Widowed/	47	17.1%	68	24.7%	138	50.2%	22	8.0%	
Educational Level	Less than High School	22	22.7%	19	19.6%	48	49.5%	8	8.2%	0.001
	High School Degree	54	10.0%	96	17.7%	356	65.8%	35	6.5%	
	Bachelor’s degree	82	10.4%	156	19.8%	461	58.6%	88	11.2%	
	Masters or PhD Degree	16	14.3%	20	17.9%	65	58.0%	11	9.8%	
Employment status	Employed (Full-time, Part-time, Self-employed)	90	14.5%	128	20.6%	342	55.2%	60	9.7%	0.001
	Unemployed (Student, Retired, Housewife)	84	9.2%	163	17.8%	588	64.1%	82	8.9%	
Has a medical history	No medical history	100	9.5%	198	18.8%	660	62.6%	96	9.1%	0.006
	Medical History	74	15.3%	93	19.3%	270	55.9%	46	9.5%	
Current physical activity	Never and rare	65	12.1%	94	17.5%	333	61.9%	46	8.6%	0.852
	1–2 times per week	64	10.3%	128	20.5%	374	60.0%	57	9.1%	
	3–5 times per week	33	11.3%	55	18.8%	176	60.1%	29	9.9%	
	Daily	12	14.5%	14	16.9%	47	56.6%	10	12.0%	

**Table A5:** Fifth attitude item according to demographic characteristics.

The Possible Harmful Effects of Physical Activity during the Pregnancy on the Fetus Are Minimal or Non-Existent										
		Strongly Agree		Agree		Disagree		Strongly Disagree		
		*N*	%	*N*	%	*N*	%	*N*	%	*p*-Value
Age	Less than 20 years	19	18.1%	45	42.9%	35	33.3%	6	5.7%	0.025
	20–29 years	140	17.1%	418	50.9%	225	27.4%	38	4.6%	
	30–39 years	69	19.2%	199	55.4%	80	22.3%	11	3.1%	
	40+ years	53	21.1%	143	57.0%	50	19.9%	5	2.0%	
Nationality	Non-Emirati	41	24.1%	88	51.8%	37	21.8%	4	2.4%	0.130
	Emirati	241	17.6%	717	52.5%	353	25.8%	56	4.1%	
Emirate	Abu Dhabi	146	19.8%	395	53.5%	175	23.7%	23	3.1%	<0.001
	Dubai	66	13.3%	278	56.0%	132	26.6%	20	4.0%	
	Northern Emirates	70	23.2%	132	43.7%	83	27.5%	17	5.6%	
Residential Area	Rural	41	22.3%	80	43.5%	59	32.1%	4	2.2%	0.017
	Urban	241	17.8%	725	53.6%	331	24.5%	56	4.1%	
Marital status	Married	223	17.7%	679	53.8%	312	24.7%	48	3.8%	0.118
	Divorced/Widowed/	59	21.5%	126	45.8%	78	28.4%	12	4.4%	
Educational Level	Less than High School	23	23.7%	43	44.3%	26	26.8%	5	5.2%	0.125
	High School Degree	92	17.0%	277	51.2%	141	26.1%	31	5.7%	
	Bachelor’s degree	144	18.3%	430	54.6%	192	24.4%	21	2.7%	
	Masters or PhD Degree	23	20.5%	55	49.1%	31	27.7%	3	2.7%	
Employment status	Employed (Full-time, Part-time, Self-employed)	129	20.8%	306	49.4%	158	25.5%	27	4.4%	0.123
	Unemployed (Student, Retired, Housewife)	153	16.7%	499	54.4%	232	25.3%	33	3.6%	
Has a medical history	No medical history	195	18.5%	569	54.0%	258	24.5%	32	3.0%	0.026
	Medical History	87	18.0%	236	48.9%	132	27.3%	28	5.8%	
Current physical activity	Never and rare	108	20.1%	276	51.3%	132	24.5%	22	4.1%	0.723
	1–2 times per week	108	17.3%	337	54.1%	160	25.7%	18	2.9%	
	3–5 times per week	53	18.1%	148	50.5%	76	25.9%	16	5.5%	
	Daily	13	15.7%	44	53.0%	22	26.5%	4	4.8%	
